# Supplementary material for: MetaRibo-Seq measures translation in microbiomes
Source: Nat Commun. 2020 Jun 29;11:3268. doi: 10.1038/s41467-020-17081-z (PMC7324362; doi:10.1038/s41467-020-17081-z)
Supplement: Supplementary file 10 — Supplementary Data 7 [file 41467_2020_17081_MOESM10_ESM.zip › File2/Confidence_High_Taxonomy/108253_out.krona.html]

Javascript must be enabled to view this page.

members
magnitude
magnitudeUnassigned
count
unassigned
taxon
rank

108253\_out

88

superkingdom
2
88

phylum
976
88

class
88
200643

171549
88
order

815
88
family

816
genus

SRS015960\_contig\_number\_6300SRS019445\_contig\_number\_2532SRS056259\_contig\_number\_11142SRS063928\_contig\_number\_432SRS100021\_contig\_number\_470SRS104400\_contig\_number\_27356SRS147139\_contig\_number\_4630
88
7

species

SRS014313\_contig\_number\_1067
28119
1

818
6

SRS015794\_contig\_number\_125SRS022713\_contig\_number\_1481SRS050299\_contig\_number\_9304SRS098073\_contig\_number\_2897SRS1041129\_contig\_number\_2030SRS893378\_contig\_number\_443
species

820
48
species

SRS011239\_contig\_number\_13093SRS012849\_contig\_number\_17617SRS013098\_contig\_number\_35701SRS014613\_contig\_number\_15295SRS015264\_contig\_number\_2552SRS016018\_contig\_number\_10264SRS016897\_contig\_number\_99SRS018427\_contig\_number\_7790SRS018541\_contig\_number\_1652SRS018575\_contig\_number\_5276SRS018836\_contig\_number\_128SRS019496\_contig\_number\_3263SRS023583\_contig\_number\_11346SRS024331\_contig\_number\_25162SRS042628\_contig\_number\_15262SRS045713\_contig\_number\_17877SRS047014\_contig\_number\_23399SRS048164\_contig\_number\_16885SRS049446\_contig\_number\_2326SRS049995\_contig\_number\_25017SRS051610\_contig\_number\_8884SRS056519\_contig\_number\_11555SRS064232\_contig\_number\_1337SRS064276\_contig\_number\_28996SRS064645\_contig\_number\_510SRS077335\_contig\_number\_1046SRS077730\_contig\_number\_57SRS097889\_contig\_number\_18604SRS098655\_contig\_number\_101SRS098717\_contig\_number\_3839SRS103987\_contig\_number\_25409SRS1041091\_contig\_number\_16370SRS1041095\_contig\_number\_1463SRS1041134\_contig\_number\_10818SRS1041137\_contig\_number\_26642SRS1041164\_contig\_number\_429SRS104975\_contig\_number\_13965SRS1054716\_contig\_number\_13532SRS1055076\_contig\_number\_9610SRS143372\_contig\_number\_7091SRS143722\_contig\_number\_14044SRS143895\_contig\_number\_39743SRS144135\_contig\_number\_26094SRS147977\_contig\_number\_568SRS148196\_contig\_number\_21413SRS149325\_contig\_number\_7238SRS150029\_contig\_number\_11081SRS893366\_contig\_number\_703

2
2292931

SRS076804\_contig\_number\_19204SRS077086\_contig\_number\_245
species

1739517
1
species

SRS054956\_contig\_number\_2265

1
2292002
species

SRS058723\_contig\_number\_5297

species

SRS142890\_contig\_number\_26861
204516
1

2
817

SRS075021\_contig\_number\_297SRS101268\_contig\_number\_28
species


SRS052078\_contig\_number\_511
species
1
2292928

species

SRS016335\_contig\_number\_29010SRS055017\_contig\_number\_8474SRS893358\_contig\_number\_12361
46506
3

1
2292920

SRS1041138\_contig\_number\_11035
species

28111
3
species

SRS020328\_contig\_number\_296SRS048981\_contig\_number\_568SRS1055043\_contig\_number\_15358

2292941
1

SRS015579\_contig\_number\_12572
species

species

SRS1055099\_contig\_number\_6028
1263049
1

821
2
species

SRS104693\_contig\_number\_619SRS1055083\_contig\_number\_1592

246787
1
species

SRS143342\_contig\_number\_27

species

SRS012273\_contig\_number\_33829
1
329854

species

SRS019286\_contig\_number\_980
2292913
1

species

SRS020271\_contig\_number\_2930SRS1041037\_contig\_number\_10005SRS893383\_contig\_number\_19972
2292924
3

1
1946089
species

SRS065176\_contig\_number\_9665
